# The evolution of Vaccination Week in the Americas

**DOI:** 10.26633/RPSP.2017.150

**Published:** 2017-12-20

**Authors:** Alba Maria Ropero Alvarez, Hannah Jane Kurtis, Lauren Vulanovic, Hayatee Hasan, Cuauhtémoc Ruiz, Elizabeth Thrush

**Affiliations:** 1 Pan American Health Organization Regional Office of the World Health Organization for the Americas Washington, DC. United States of America Pan American Health Organization, Regional Office of the World Health Organization for the Americas, Washington, DC, United States of America. Send correspondence to Alba Maria Ropero Alvarez; 2 World Health Organization World Health Organization Geneva Switzerland World Health Organization, Geneva, Switzerland.

**Keywords:** Immunization programs, mass vaccination, global health, health diplomacy, Americas, Programas de inmunización, vacunación masiva, salud global, diplomacia de la salud, Américas, Programas de imunização, vacinação em massa, saúde global, diplomacia em saúde, Américas

## Abstract

*This report covers the background and evolution of Vaccination Week in the Americas (VWA), an initiative that started as a coordinated response to a 2002 measles outbreak in Colombia and Venezuela, and evolved into the model for other regions and World Immunization Week (WIW)*.

*VWA focuses on the work of national immunization programs, with special efforts to reach the unreached. This paper offers examples of how countries have leveraged VWA to implement a diverse array of vaccination activities, strengthening overall health services by integrating with other preventive health interventions, and bolstering “Pan-Americanism” and health diplomacy*.

*The opportunities offered by this global initiative were clearly demonstrated in April 2016 when the successful global switch from the trivalent oral polio vaccine to the bivalent vaccine was synchronized with WIW. Going forward, VWA and WIW can help close the gaps in access to immunization and other health services, contributing to achieve universal health coverage*.

## RESUMEN

Este informe incluye los antecedentes y la evolución de la Semana de Vacunación en las Américas, una iniciativa que comenzó como una respuesta coordinada a un brote de sarampión en Colombia y Venezuela en el 2002, y evolucionó hasta convertirse en modelo para otras regiones y para la Semana Mundial de la Inmunización.

La Semana de Vacunación en las Américas se centra en el trabajo de los programas nacionales de inmunización, con esfuerzos especiales para llegar a las personas que todavía no estén cubiertas. En el presente trabajo se dan ejemplos de cómo los países han aprovechado la Semana de Vacunación para realizar diversas actividades relacionadas con la vacunación, fortalecer los servicios de salud en general mediante la integración con otras intervenciones preventivas y fomentar el “panamericanismo” y la diplomacia en torno a la salud.

Las oportunidades que ofrece esta iniciativa mundial quedaron claramente evidenciadas en abril del 2016 con el éxito obtenido en la sustitución de la vacuna oral trivalente contra la poliomielitis por la vacuna bivalente, sincronizada con la Semana Mundial de la Inmunización. En el futuro, la Semana de Vacunación en las Américas y la Semana Mundial de Inmunización pueden ayudar a subsanar la brecha en el acceso a la inmunización y otros servicios de salud, y contribuir a alcanzar la cobertura universal de salud.

## RESUMO

Este artigo narra os antecedentes e a evolução da Semana de Vacinação nas Américas (SVA), uma iniciativa que nasceu da resposta coordenada ao surto de sarampo na Colômbia e na Venezuela em 2002 e evoluiu como um modelo para as outras regiões e para a Semana Mundial da Imunização.

A SVA põe ênfase no trabalho dos programas nacionais de vacinação e, principalmente, nos esforços para sealcançar quem ainda está inalcançado. São ilustrados exemplos de como os países têm aproveitado a SVA para implementar várias atividades de vacinação, fortalecendo os serviços de saúde em geral ao integrá-la a outras intervenções preventivas e reforçando o pan-americanismo e a diplomacia da saúde.

As oportunidades criadas por esta iniciativa global ficaram bem evidentes em abril de 2016 com a bem-sucedida transição em nível mundial da vacina oral contra poliomielite trivalente à bivalente, em sincronia com a Semana Mundial da Imunização. Prosseguindo adiante, a SVA e a Semana Mundial da Imunização podem contribuir para sanar as lacunas no acesso à imunização e a outros serviços de saúde e alcançar a cobertura universal de saúde.

Every April during Vaccination Week in the Americas (VWA), the countries and territories in the Region of the Americas celebrate the unique power of vaccination to prevent disease. For the past 15 years, VWA has been one of the flagship initiatives of the Pan American Health Organization (PAHO), vaccinating an estimated 686 million individuals through the activities conducted under its framework (1).

VWA initially grew out of a proposal by the Ministers of Health of countries in the Andean subregion following a 2002 measles outbreak in Venezuela and Colombia (2). The ministers called for a harmonized, annual, international vaccination effort as a means of preventing future outbreaks. The proposal evolved and gained momentum in the succeeding months, and with PAHO support, 19 countries and territories participated in the first VWA in 2003 (3). That September, the budding initiative was endorsed by PAHO Member States during the 44th Directing Council, setting the political mandate for the implementation of VWA in future years (4).

## STRATEGIC OBJECTIVES

**FIGURE 1.**
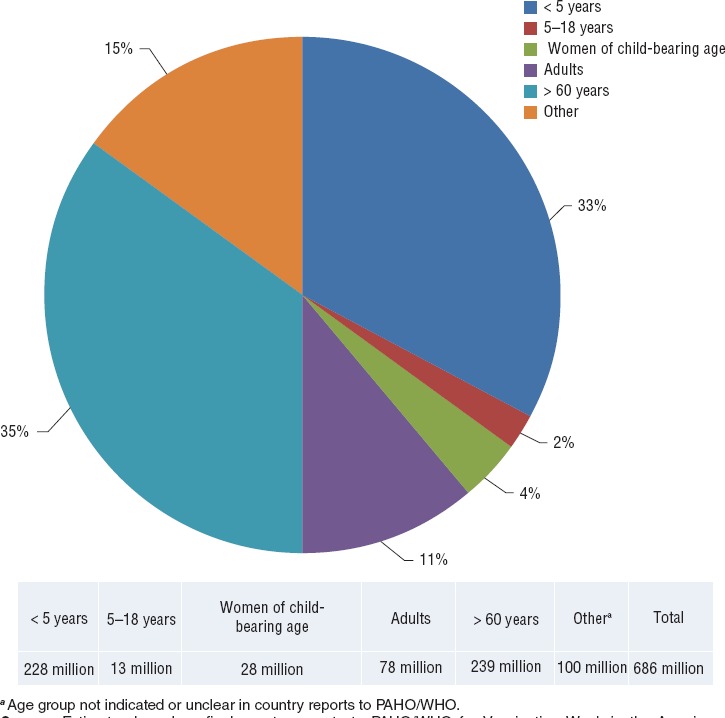
Estimated number of people vaccinated, by age group, as part of VaccinationWeek in the Americas (VWA), 2003–2017

***Source:*** Estimates based on final country reports to PAHO/WHO for Vaccination Week in the Americas, 2003–2017.

The 2002 measles outbreak in Venezuela and Colombia was a stark reminder that high vaccination coverage at the national level can obscure the presence of low coverage pockets at the district and municipal levels, giving a single imported case the power to trigger a large outbreak among a susceptible population (5). In response, one of the core, strategic objectives for creating VWA was to focus on strengthening immunization programs, reaching populations living in vulnerable situations: those with limited access to regular health services, especially populations in border and rural areas, urban fringes, indigenous communities, and other high risk groups. By “reaching the unreached,” countries are able to use VWA to reinforce coverage by their national immunization programs and to advance health equity. Additional VWA strategic objectives are: targeting individuals across the life course (Figure 1); highlighting the work of national immunization programs in the media; keeping immunization on the forefront of political agendas; using the initiative as a platform to integrate other preventative health interventions; and promoting cooperation and collaboration among countries, public health agencies, and partners.

## IMPLEMENTATION

From its inception, VWA was designed to be a flexible initiative and a tool for strengthening national immunization programs. Each year, countries select their respective VWA activities based on their current national health priorities (6). Countries also shoulder the vast majority of implementation costs, reflecting widespread national commitment to the initiative.

In general, national VWA activities can be broadly categorized as efforts to:(i) sustain vaccination achievements;(ii) complete the unfinished agenda for prevention and control of vaccine-preventable diseases; (iii) tackle new challenges in vaccine introduction and impact assessment; and (iv) strengthen health services for effective vaccine administration. These four areas are the strategic lines of action of the Regional Immunization Action Plan (7), an adaptation of the WHO Global Vaccine Action Plan (GVAP; 8) and the guiding policy document for immunization in the Americas for 2015 – 2020. Country activities during VWA have been implemented with regard to these four lines of action, including:

(i) **Sustain the achievements**
  – Indiscriminate vaccination against poliomyelitis, plus mass campaigns against measles, rubella, and congenital rubella syndrome (CRS) have been used to maintain the elimination of polio (9) and elimination of rubella and measles (10) in the Americas despite the continued global circulation of these viruses.
(ii) **Complete the unfinished agenda**
  – Efforts to start, update, and complete national childhood immunization schedules, commonly targeting those < 5 years of age, have used house-to-house vaccination brigades mobilized via air, water, and land; vaccination posts in shopping centers, community plazas, soccer stadiums, and other public areas; and institutional vaccination in health centers with extended hours. School-based efforts have also delivered vaccines to older children.
  – Vaccination against seasonal influenza in high risk groups in the Southern Hemisphere, since VWA coincides with this influenza season.
  – Preventing maternal and neonatal tetanus through vaccinating women of childbearing age with tetanus-containing vaccines.
  – Outreach for groups at increased occupational risk (e.g., farmers, fishermen, construction workers, teachers, tourism/border control officers,health workers) to prevent hepatitis B, influenza, and tetanus.
(iii) **Tackle new challenges**
  – Introduction of new vaccines (e.g., human papillomavirus, pneumococcal, and rotavirus) into national vaccination schedules, including staff training and community awareness efforts.
(iv) **Strengthen health services**
  – Integration of vaccination with other public health interventions, such as: vitamin A supplementation; deworming; HIV testing; breastfeeding promotion; and screenings for diabetes, hypertension, and other non-communicable diseases (11).
  – With Zika emergence in 2015, countries used the VWA platform to raise public awareness of mosquito-borne diseases and elimination of breeding sites as a means of preventing Zika as well as dengue, chikungunya, and yellow fever (12).
  – Education for health workers on vaccine-related topics, outreach to health professional students, and sensitization of teachers and parents to the importance of children being fully immunized.

**TABLE 1.** Estimated cumulative number of vaccines administered by antigen as part of Vaccination Week in the Americas (VWA), 2006–2017

Vaccine	Estimated number of doses
Influenza	296 884 930
Polio	109 163 189
Measles	69 130 983
Diphtheria–Pertussis–Tetanus	31 413 900
Hepatitis B	14 857 484
Yellow fever	14 177 613
Pneumococcal	6 383 336
Pentavalent^a^	5 295 722
Rotavirus	3 464 394
Bacillus Calmette-Guérin	1 965 438
Varicella	1 869 637
Human Papilomavirus	1 195 970
Haemophilus influenza type b	491 322
Other (not specified)	3 218 306

***Source:*** Estimates based on final country reports to PAHO/WHO for Vaccination Week in the Americas, 2006–2017.

### VWA results

Given the evolution of VWA, detailed data are not available for its first few years. However, based on final country reports to PAHO/WHO, the estimated cumulative number of vaccines administered, by antigen, as part of VWA efforts (2006–2017) are available in Table 1. Mass campaigns in large countries, such as influenza in Brazil, account for a substantial proportion of vaccines administered during VWA; however, intense efforts to reach and vaccinate remote, small communities cannot be overlooked and are critical to the initiative’s main purpose.

## COMMUNICATION AND SOCIAL MOBILIZATION

VWA has provided a key opportunity for countries to engage with the general public on a range of immunization-related topics and to help individuals and communities understand that vaccination is a basic right at all stages of life. Each year, PAHO proposes an overarching Regional campaign theme, one that is expected to resonate with the public. Themes often relate to current events or interests, such as the World Cup, the Olympic Games, or to pop culture. Past slogans have included “Boost your power! Get vaccinated!;” “Vaccination: Your best shot;” and “Go for the gold! Get vaccinated!” Regional campaign materials reflect the theme and can be adapted by each country. Celebrities have lent their voices as champions of health, promoting vaccination, and helping PAHO and public health authorities to reach broader audiences. Past champions have been the Jamaican olympian, Usain Bolt, and the Brazilian football star, Ronaldo; star entertainers and musicians, such as Ricardo Montaner and Don Francisco; and beloved children’s characters, namely, the Sesame Street puppets and “El Chapulín Colorado” (1, 13–15).

Social media has played an important part in VWA activities since 2011, giving PAHO and countries access to the general public while advocating for immunization, dispelling myths, and strengthening collaboration with partners. Every year, social media messaging for VWA is programmed on PAHO regional and country social media accounts; messages are also shared with key partners to reach a larger audience. Messaging is geared toward caregivers, health care workers, decision makers, and the general public, and aims to: (a) highlight the importance of vaccination in protecting the population’s health;(b) encourage people to get themselves and their family members vaccinated; (c) motivate people to show their support for vaccination; (d) raise awareness of the work PAHO does to promote vaccination in the Region; (e) recognize the outstanding public health achievements brought about by vaccination; and (f) advocate for continued support of a strong Expanded Program on Immunization (EPI). It is increasingly important to spread positive vaccination messages through social media, as this is a channel commonly used by anti-vaccination groups.

Countries also develop their own national VWA communication activities and mass media campaigns using television, radio, and print media to increase immunization awareness and to highlight its achievements. Many countries also focus on sensitization sessions and community fairs to educate parents and the general public on vaccination issues (1, 13–15).

## INTERNATIONAL COORDINATION AND HEALTH DIPLOMACY

Disease transmission knows no borders, and for that reason, international collaboration in fighting vaccine-preventable diseases is of utmost importance. Over its tenure in the Region, VWA events have facilitated cross-border cooperation and provided the opportunity to keep immunization on the forefront of political agendas.

Each year, numerous celebrations are held throughout the Region to launch VWA, with a special emphasis on bi- and tri-national events. These activities often include the participation of high level authorities, such as presidents, first ladies, celebrities, governors, mayors, and local leaders, as well as the Director of PAHO and representatives from other United Nations agencies, non-governmental organizations, and civil society. Media coverage of VWA celebrations gives visibility to the benefits of vaccination and the work of national immunization programs, and puts the focus on the needs of the host communities (6). In border areas, organizing a VWA launch requires collaboration between the government ministries and local authorities on both sides—international collaboration purely in the name of public health and Pan-Americanism.

Some examples of bi- and tri-national VWA launches include events among Argentina and Chile; Argentina and Uruguay; Bolivia and Peru; Argentina, Bolivia, and Paraguay; Brazil, Colombia, and Peru; Colombia and Panama; Ecuador and Peru; Costa Rica and Panama; Guyana and Suriname; Guyana and French Guiana; Honduras and El Salvador; Honduras and Guatemala; Nicaragua and Costa Rica; Guatemala and Mexico; and Mexico and the United States. A special opportunity was presented in 2013 when the Regional VWA launch was held in the Adjacency Zone between Belize and Guatemala (1, 3, 5, 13, 14).

VWA has also become a useful tool through which countries can collaborate to reach Regional and sub-regional vaccination goals. For example, though the 2002 outbreak of measles and rubella was effectively contained (16), during the next several years many countries continued conducting measles follow-up campaigns to raise their national coverage rates. Moreover, during VWA 2008, the countries of Central America and Mexico ran simultaneous measles follow-up campaigns to raise coverage throughout the area at the same time (17).

Overall, while the work of national immunization programs in the Americas continues year round, VWA has provided a highly visible platform from which countries can make the final push toward disease elimination. The Region of the Americas was the first in the world to be declared free of endemic transmission of rubella and CRS in 2015; this achievement was followed in 2016 by the Region being the first declared free of endemic transmission of measles (10). Likewise, VWA has provided a critical opportunity to help countries maintain control of other vaccine-preventable diseases.

## EVOLUTION OF A GLOBAL MOVEMENT

**FIGURE 2:**
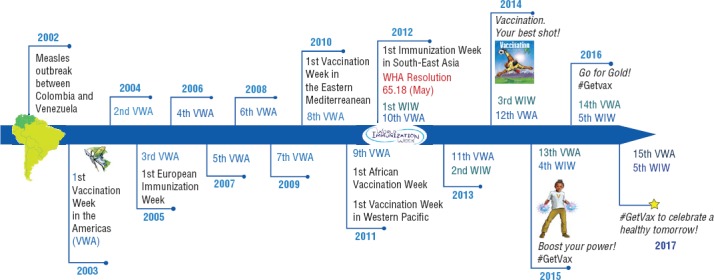
Timeline of Vaccination Week in the Americas (VWA) and other sister vaccination week initiatives, 2002–2017

***Source:*** Comprehensive Family Immunization Unit, Pan American Health Organization, Washington DC, United States, 2017.

The successful experiences with VWA in the Americas have served as a model for other WHO Regions to establish and develop their own sister initiatives (Figure 2). The first to follow suit was the European Region, creating European Immunization Week in 2005. In 2010, the Eastern Mediterranean Region launched its first vaccination week, followed by the African Region and Western Pacific Region in 2011, and the South-East Asia Region in 2012 (5, 18).

Like VWA, the priorities of the other Regional vaccination weeks are tailored to meet the needs and activities of their Member States. Some regions focus on vaccination campaigns, while others, such as the European Region, use this week to communicate with the general public in an effort to increase acceptance of and demand for vaccination, and to counteract the messages of anti-vaccination groups. As a global movement, Vaccination Week reached a milestone in 2012 when the Ministers of Health of 194 WHO Member States endorsed Resolution WHA65.18 at the 65th World Health Assembly, establishing the last week of every April as World Immunization Week (WIW) (5).

Since 2012, WIW has served as an overarching framework linking together all of the regional vaccination weeks. Guided by the objectives of the WHO GVAP (8), it emphasizes the importance of expanding access to immunization, achieving the Sustainable Development Goals (19), and aiming to keep immunization a global health priority, while promoting its role in human and economic development (20).

Using WIW as a platform to advance GVAP goals is of particular importance considering that globally, there are an estimated 19.4 million children, many of whom live in low-income countries and are unprotected because they have not received a complete, recommended national vaccination schedule (21). Since one of the primary goals of GVAP is equitable access to immunization (8, 21), WIW has become a key tool for closing the gaps (19), bringing countries closer to GVAP goals by 2020 (8) and Sustainable Development Goals by 2030 (22).

Additionally, WIW uses recommendations from the WHO Strategic Advisory Group of Experts (SAGE) on Immunization to plan annual campaigns. WIW can, therefore, serve as a platform for implementing SAGE recommendations. This was done successfully during WIW 2016 with the globally-synchronized switch from the trivalent oral polio vaccine to the bivalent vaccine. In all, 155 countries worldwide, including 36 countries in the Americas, made the switch—taking full advantage of the unique spotlight cast by WIW and the celebration of the eradication of type 2 poliovirus.

### Conclusion

Vaccination is one the most successful and cost effective interventions in public health (23); VWA and WIW are yearly opportunities to celebrate its power to prevent disease and to encourage governments to continue guaranteeing vaccination as a right. VWA in particular highlights the strategic partnerships that have helped the Region continue to be a global leader in disease control and elimination. As a social mobilization campaign, relevant themes and the use of diverse media have allowed PAHO, its partners, and Member States to give visibility to the importance of immunization and communicate with key audiences, building public confidence in vaccination, and countering the emerging messages of anti-vaccination groups.

Vaccination helps prevent an estimated 2–3 million deaths and many more debilitating illnesses each year. The effects of healthier populations have educational and economic implications, including a reduction in health care costs, less school and job absenteeism, and increased productivity and quality of life (23). The VWA goal of vaccinating underserved populations helps expand these benefits to all people, increasing equality and bringing countries closer to achieving universal health coverage.

Looking to the future, WIW, VWA, and the other regional vaccination weeks will continue to offer a critical platform to harness political will, increase public demand for vaccination, and close the current gap in access to immunization and other health services.

### Acknowledgements

The authors would like to acknowledge the efforts of the countless health care workers throughout the Region without whom Vaccination Week in the Americas would not be possible. The authors also would like to recognize the generous support from partners and agencies that have helped make VWA a success.

### Disclaimer

Authors hold sole responsibility for the views expressed in the manuscript, which may not necessarily reflect the opinion or policy of the *RPSP/PAJPH *and/or PAHO.
